# Retention and future involvement in the American Kennel Club Junior Showmanship Program, a youth dog breed conformation competition

**DOI:** 10.3389/fvets.2022.871914

**Published:** 2022-09-23

**Authors:** Hannah L. Loonsk, Dana L. Clarke, Carmen Battaglia, Cynthia M. Otto

**Affiliations:** ^1^University of Pennsylvania School of Veterinary Medicine, Philadelphia, PA, United States; ^2^Department of Clinical Sciences and Advanced Medicine, University of Pennsylvania School of Veterinary Medicine, Philadelphia, PA, United States; ^3^American Kennel Club, New York, NY, United States; ^4^Penn Vet Working Dog Center, University of Pennsylvania School of Veterinary Medicine, Philadelphia, PA, United States

**Keywords:** retention, sports, volunteer, mentorship, dog, survey, youth development, show

## Abstract

Similar to other organizations that encourage positive youth development, the American Kennel Club (AKC) created the Junior Showmanship program to develop skills and pave a path for the next generation of competitors in canine conformation. Although participants age out of the Junior Showmanship Program when they turn eighteen, the hope is that Juniors will continue to be active in dog sports into adulthood. Females are overly represented in all age groups in this survey and in current participation. Although both males and females most commonly stopped participating at age 17 or 18, males had a significantly higher drop out at age 15. Further study is warranted to investigate factors specifically pertaining to male participation. A strong Belief System Model which accounted for positive responses associated with camaraderie and mentoring during the showmanship program was significantly (p=0.01) associated with commitment, member interest and active participation as an adult. In addition, success in the show ring was associated with future involvement in conformation or dog sports. Thus, both perceived achievement and mentorship by adult “volunteers” were associated with continued participation.

## Introduction

Positive youth development (PYD) programs have received attention from the scientific literature and public policy since the 1960's (1). These programs aim to provide youth with the resources to develop into successful adults through positive relationships with adults, challenging experiences and building skills (1). The 4-H (a United States Cooperative Extension Service youth development program; https://4-h.org/) has proactively developed their programing to reflect the elements of PYD programs (such as “positive relationship with a caring adult, a safe emotional and physical environment, an inclusive environment, engagement in learning, opportunity for mastery, opportunity to see oneself as an active participant in the future, opportunity for self-determination and opportunity to value and practice service to others”) (2). Many extracurricular activities directed at youth actively address the elements of these PYD, other programs have incorporated these elements through natural evolution.

In 1932, the Westbury Kennel Association offered the first “Children's Handling Class.” The goal of this innovative new competition was to provide a distraction for the restless children accompanying adults at dog shows. It had the added benefit of increasing future involvement in the dog show community. The class was judged only on the ability to present the dog “quietly, proficiently and to its best advantage. “The first judges were local celebrities, who were later replaced by AKC judges. In 1951, the Children's Handling Class was renamed “Junior Showmanship” and it became a recognized AKC event in 1971 (3). In 1998, the AKC began to offer scholarships for Juniors and broadened its definition of Juniors to include performance events. Children between 9 and 18 years of age are eligible to participate in Junior Showmanship, a unique combination of competition and PYD. Table 1 illustrates the breakdown of divisions within the program. Winners from each class compete for Best Junior and Reserve Best Junior. Junior Showmanship participants age out when they turn eighteen. The involvement of adults in Junior Showmanship is major factor that contributes to the positive environment for participants. Adults must also fill logistical and financial support roles in addition to other guidance, training, or emotional support. Adult individuals should be designated as “volunteers” because of the dedication of time and effort associated with supporting a Junior (4).

**Table 1:** Age breakdown of divisions for each class within the Junior Showmanship program.

**Novice**	**Open**		
**Class**	**Age group**	**Class**	**Age group**
Junior	9 and under 12	Junior	9 and under 12
Intermediate	12 and under 15	Intermediate	12 and under 15
Senior	15 and under 18	Senior	15 and under 18

There are limited studies that address short-term retention in PYDs and youth sports (5–8). There are no current studies evaluating success or risk factors for children who cease to participate in or “dropout” of AKC dog sports. Historical trend data provided by the AKC show a slow decrease in program participation between the years 2000 and 2013 (9). This survey aimed to collect data on factors that could predict Junior “success in the ring,” perception of fun, and program dropout. It also investigated gender discrepancies, patterns of educational achievement, mentorship efficacy, and continued involvement in conformation and dog sports after “aging-out.”

We hypothesized that retention in the program is positively correlated with future activity as an adult exhibitor or breeder.

## Methods

The study was reviewed by the University of Pennsylvania Institutional Review Board and determined to be exempt from review requirements. Participants were recruited through social media, including outreach to current AKC delegates (10). Data collection occurred through an anonymous internet-based cross-sectional survey of individuals previously involved in the AKC Junior Showmanship program, participants consented to use of the data for research purposes. The survey was initiated on 29 January 2012 and ended on 11 July 2012. In order to evaluate the long-term impact of participation in the program, we limited survey participation to individuals who were at least 10 years out of the Junior Showmanship program.

The 50-question survey was divided into three segments (questionnaire available in Supplementary materials): (1) questions pertaining to the time period of Junior participation, (2) questions pertaining to activities within the last 5 years, and (3) questions pertaining to the present. The survey collected details about the responden's age, gender, and education, as well as the respondent's family, other socio-economic and environmental factors, and any scholarships received through the AKC. The survey presented predefined choices only. No write-ins or short answer comments were analyzed. Respondents were allowed to skip questions.

**Figure 1:**
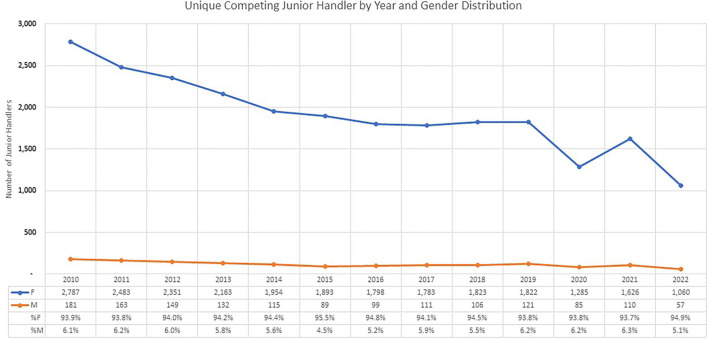
Historical data from the AKC showing distribution of males (red line) and females (blue line) between 2010 and 2022. The data table represents the absolute number and relative percentage of females and males competing per year.

Historical data from 619 all-breed clubs that offered Junior showmanship competitions (2012 and 2022) were collected directly from the AKC (Figure 1).

Dropout was defined as ceasing to participate in the Junior Showmanship program prior to aging out at 18. Dropout percentage was calculated by dividing the number of survey respondents who ceased to participate prior to aging out by the total number of survey respondents. Data were reviewed visually, and the frequency of responses reported. Data were evaluated for normality by the Shapiro-Wilk test. Normally distributed data were reported as the mean and standard deviation, whereas non-parametric data were reported as median. For age distribution, individuals were assigned a category based on 10 year increments, with the exception of 18–20 and above 71 years of age. Two types of inferential statistics were used in this report: chi-squared analyses and correlation analyses. Inferential Statistics were used to reach conclusions that extends beyond the immediate data (descriptive statistics) and Chi-Squared analyses was used to show differences between groups or categories observed in the data. A *p* value of < 0.05 was considered statistically significant. A Belief System Model (BSM) (11); was developed to measure the camaraderie amongst the Juniors, based on responses related to: their supporting network, friends reaction, how friendly the judges were, how much guidance the judges provided, how many friends at show, if they were attracted to the AKC because: their friend was a Junior, if their parent encouraged them, or if they were attracted by someone else (alpha = 0.6267 very stable). The BSM was then tested for correlation to the following variables: commitment [measures the current commitment to the AKC based on the number of meetings attended, or served as an officer, AKC delegate (10), AKC judge and AKC eligibility], member interest (current interest in the AKC based on the number of memberships to clubs and their AKC eligibility. Clubs include: parent club, merit program, all breed member, and other dog club), AKC recency (the extent of recent involvement in AKC shows, based on: the number of dogs handled in conformation in the last 12 months and the number of champions finished and handled in the last 5 years), social economic status, education, beliefs about judge indifference, success in the show ring as a Junior (i.e., placement in the competition), how fun the experience was, the inverse of how stressful it was, gender, age, decade of competition, receipt of scholarship and years spent showing (see Supplemental Table 1). Correlations between BSM and how it relates to involvement as a Junior, involvement now and dedication to the sport are presented in Supplemental Table 2.

## Results

Of the 1,346 surveys started, 329 were ineligible due to < 10 years since they competed as a Junior and 476 failed to complete the survey. Of the 870 eligible and complete surveys, the median age was 39.3 years old. Most of the respondents who took the AKC survey were between the ages of 21–60 (Table 2). The survey respondents were 84.9% female and 15.1% male. In this survey, males are over-represented compared to the 2010–2021 AKC statistics, where males represent 5.8% of the total participants (Figure 1). Participants in the Junior Showmanship program between 1992 and 2001 made up 34.5% of survey respondents, while participants from 2002–2012 made up 24.4% of survey respondents. Finally, 41.1% of survey respondents participated prior to 1992. Most Juniors showed their own dog (*n* = 710, 52.63%), followed by their family's dog (*n* = 512, 37.95%), followed by other's dogs (*n* = 367, 27.21%). Of all survey respondents, 78.2% of respondents began the program in the Novice Junior class and 86.8% finished in the Open Senior class. When respondents were asked at what age they participated in their last Junior Showmanship class, 18 years old was the most frequent response. However, only 36.7% of the respondents were 18 years old the last time that they participated. 63.3% of respondents dropped out of the program prior to aging out at 18 years old. The proportion of males and females dropping out each year was similar, except significantly (*p* < 0.05) more males dropped out at age 15 (see Table 3). The number of years of participation are in Table 4.

**Table 2:** Frequency of age groups of AKC Junior survey respondents.

**Age group**	**Frequency**	**Percent**
18–20	13	1.49%
21–30	264	30.34%
31–40	246	28.28%
41–50	178	20.46%
51–60	132	15.17%
61–70	32	3.68%
71–102	5	0.57%

**Table 3:** Age at which male and female survey respondent last participated in the Junior Showmanship Program as percentage of respondents.

**Age**	**9**	**10**	**11**	**12**	**13**	**14**	**15**	**16**	**17**	**18**
female	0.14	0.83	0.55	1.93	3.04	2.62	4.97	14.78	32.87	38.26
male	0.79	0.00	2.38	2.38	2.38	3.97	11.11*	19.05	30.16	27.78

*significantly more males ended participation at age 15.

**Table 4:** Number of years in the show ring.

**Number of years**	**Frequency**	**Percent**
0	14	1.64%
1	16	1.88%
2	54	6.34%
3	71	8.33%
4	106	12.44%
5	102	11.97%
6	104	12.21%
7	112	13.15%
8	161	18.90%
9	112	13.15%

Of respondents 56.1% held at least a 4-year college degree. When asked about previous or current jobs, 53.7% of survey respondents stated that they had held a job related to dogs, with 12.9% working in the veterinary medical field. Other reported jobs included animal research, the dog food industry, the AKC, professional handling, animal advertising, animal law, and dog training. Scholarships from the AKC were awarded to 11.9% of respondents and 41.7% of these scholarship recipients reported that the scholarship was for 1 year.

At the time of the survey, 74.3% of respondents owned three or more purebred dogs. In the prior 5 years, 67.7% of respondents had bred one or more litters and 86.9% of these individuals had bred at least one AKC Champion in the last 5 years. 37.2% of respondents participate in the AKC's Breeder of Merit program (12), 82.7% handle conformation dogs in the last 12 months, and 66.1% belong to a Parent Breed Club. In the last 5 years, 176 respondents served as Junior Showmanship judges and 122 respondents served as conformation judges. 655 respondents identified themselves as breeders and/or exhibitors and 308 self-identified as professional handlers.

Respondents were asked to rank the significance of mentorship they received from various adults within conformation competition. 53.7% of respondents reported that they received substantial support from their mother, 47.1% received substantial support from a friend, and 46.3% reported being significantly supported by a professional handler. 32.7% of respondents indicated that they had a sibling also participating in the Junior Showmanship program and 78.6% of respondents reported to have friends participating in the Junior Showmanship program. 66.5% of respondents reported becoming interested in Junior Showmanship because they were already traveling to shows with parents or friends. 52.7% of respondents reported that their parents were exhibitors and 51.5% reported that their parents were breeders.

There was a highly significant relationship between years spent in show with parental encouragement, *p* < 0.01) and family support (mentor), *p* < 0.01. Four hundred and ten Juniors had parental encouragement, while 442 did not. For the Juniors who spent 8 or 9 years in show, most of them had parental encouragement. Juniors were more likely to indicate family support and mentorship the more years they spent in show.

There was a highly significant relationship between years spent in show and having very high success in the ring during participation, *p* < 0.01. Around a third of Juniors fall into the “very high success category” (*n* = 366). Out of the 366 Juniors with very high success, most of them spent >4 years in show, with 91 of them spending 8 years in show.

There was a statistically significant relationship between success in the ring as a Junior and having a friend (*p* ≤ 0.05) or mother (*p* ≤ 0.05) as a mentor. There was no relationship between a father as a mentor and success in the ring and sibling mentorship was inversely related to success in the ring (*p* ≤ 0.01). Furthermore, success in the show ring was significantly (*p* = 0.01) correlated with future member interest (memberships to clubs and AKC eligibility- recent) (*r* = 0.5220) and commitment (recent- meetings, delegates, etc) (*r* = 0.4648).

Respondents were asked to qualify which aspects of Junior Showmanship made it “fun,” 85.8% of respondents answering this question strongly agreed with the choice “working with a dog” and 77.5% of respondents strongly agreed with “feelings of accomplishment.” Finally, 63.5% of respondents strongly agreed with the statement “winning is fun.” Success in the show ring as rated as “very high” by 44.5% of respondents and 47.7% qualified their success as “moderate.” The majority (77.4%) of respondents agreed or strongly agreed with the statement “The judges were friendly.” Over half, (56.9%) of the respondents agreed or strongly agreed with the statement “The judges provided guidance,” whereas 80.2% of the respondents felt that “the judges were business-like.”

The Belief System Model (BSM) was correlated with Member Interest (highly significant, *p* = 0.01 and positively correlated 0.4001), commitment (highly significant, *p* = 0.01 and positively correlated 0.3575) and success as a Junior (highly significant, *p* = 0.01 and positively correlated 0.5275). Several weak correlations were highly significant (*p* = 0.01) and positively correlated with BSM, including AKC Receny (0.1438) experience of fun (0.2238), stress (reverse scored, thus lower stress was associated with higher BSM) (0.2411) and show years (0.1639); whereas education level (−0.1285) feelings about judges' indifference (−0.2372) and competition decade (−0.2433) were inversely correlated.

The majority of respondents were female. Gender was weakly but highly significantly (*p* = 0.01) and negatively correlated (−0.1503) to the experience of fun, (meaning that the greater the fun experienced, the more likely respondent was female), member interest (−0.1556; women were more likely to be interested members in adulthood) and AKC recency (−0.0682; women were more likely to be more involved in the AKC as an adult).

## Discussion

The AKC Juniors program features several key characteristics that are found in positive youth development programs, including a positive relationship with a caring adult, a safe emotional and physical environment, engagement in learning, opportunity for mastery, and opportunity to see oneself as an active participant in the future (2).

The majority of survey respondents confirmed their ongoing participation as AKC Breeders of Merit (12), AKC delegates (10), AKC judges of all venues, AKC employees and board members, dog club officers, veterinarians, registered handlers (RHP members), and exhibitors, this involvement was associated with success in the show ring and a positive BSM, highlighting the importance of mentorship and camaraderie in developing a long term affiliation with conformation and dog sports. In addition to the benefit to youth development, involvement of youth provides the community with the next generation of leaders and mentors.

A multitude of factors influenced children participating in AKC events. As defined by the inclusion criteria, all survey respondents had stopped competing in Junior Showmanship at least 10 years prior to participating in the survey. The average age of respondents indicates that most had been out of the Junior Showmanship Program even longer (see Table 2).

Based on the survey data, the Junior Showmanship program retention rate of survey respondents was 36.7%. The dropout rate, defined by cessation of participation prior to the age of eighteen, was 63.3%, but it is worth noting that 32.4% of respondents participated in the program until the age of seventeen. This dropout rate alone does not provide definitive information on further participation in AKC events because 82.7% of survey respondents reported having handled a dog in conformation in the last 12 months. Dropping out of the Junior Showmanship program does not necessarily correlate with cessation of participation in AKC events. Some individuals who stopped participating in Junior Showmanship prior to aging out continued to participate in AKC Conformation for at least 10 years. This survey was limited in its ability to capture people who dropped out of the Junior Showmanship program and are no longer in any way connected with the AKC.

The survey data highlight a discrepancy in the proportion of male and female participation in the Junior Showmanship Program, although the proportion of males responding to the survey is higher than current male participation in Junior Handling. The percentage of males that stopped participating at age 15 was higher than the percentage of females that stopped participating at that age. Competitive dog events and horseback riding are among the only sports where men and women are in direct competition. The sport (including conformation) of dogs, like that of horses, offers the rare opportunity for direct competition between men and women, but young boys frequently struggle with losses to female peers (13). A 1987 paper by Eder and Parker describes how, as early as elementary school, extracurricular activities have been shown to potentiate gender discrepancies during the early development of values. Boys are taught to be tough, competitive, and achievement-oriented. In contrast, girls are encouraged to be nurturing and develop useful skills. Emphasis of these traditionally accepted gender roles within structured activities leads to their social persistence (14). In 1983, Gill et al. (15) demonstrated that young boys weighed achievement and status more heavily than young girls, when asked about factors relating to continued participation in a questionnaire (15). Girls were more likely to have a high rating for the experience of fun during showing, but there was not a detectable association with gender and “success in the ring” (i.e., placing in the competition). In other youth directed programs that involve animals (e.g., Pony Club, 4-H) (16); (https://arr.news/wp-content/uploads/2021/02/PCA-Surveys-Boys-about-Pony-Club-feb-2021-1.pdf), girls are also overrepresented. This may represent a stronger affinity for animals, stronger female mentorship, or fewer options for programming for young girls (e.g., organized sports). In contrast to animal based youth sports and organizations, boys are over-represented in traditional organized sports, even from a very young age. In 2012, the percentage of boys in the US between 6 and 12 participating in organized sports (49.1%) far outnumbered girls (33.5%) (https://www.aspenprojectplay.org/youth-sports/facts/participation-rates). A Canadian study of involvement in high school sports identified adolescents' sex (males > females), parental involvement, best friends' participation in sports and family income as significant predictors of participation (17). Parental support (particularly maternal for girls and paternal for boys) and peer support are important factors for teen involvement in sports or physical activities (18, 19).

By definition, participation in the Junior Showmanship Program is limited to minors. The majority of these children are not yet able to drive, so assistance is necessary to overcome even just the first hurdle of transportation. Adults also play a crucial role in supporting the Junior and dog financially. In addition to these roles, it is important to consider the adult's role in providing guidance and emotional support. Respondents were prompted to qualify the mentorship that they received during their participation in the Junior Showmanship Program. The data from this survey show a diverse mix of adults supporting Junior participation. The most common supporting adults were reported to be mothers, siblings, friends, and professional handlers. Support of friends and mothers was associated with “success in the ring.” The majority of respondents became interested in Junior Showmanship because they were already traveling to shows with parents or friends.

The data collected in this survey emphasizes the importance of mentorship and parental support. The existing literature (4, 20), suggests that adult involvement increases when the values of the volunteer are reflected in the opportunity to provide support and assistance. Values frequently appreciated by adult volunteers include self-development, good sportsmanship, social skills, responsibility, and time management (4). Combined, these data support the importance of involving adult volunteers (e.g., parents, family friends, professional handlers, or breeders) to mentor children in developing good sportsmanship, social skills, responsibility, and time management. Additional incentives for adult volunteers could include reduced entry fees or a program badge similar to the Breeder of Merit badge offered to adult exhibitors or breeders who support the future of conformation and dog sports through Junior mentorship. In 2008, Meier and Stutzer elaborated on several extrinsic reasons for volunteering including (1) Investment in human capital (i.e., to raise future earnings), (2) Investment in social network (i.e., to establish social contacts in order to increase business, get employment, or gain future material reward, and (3) To gain social approval (i.e., prestige, increase in social standing) (21). It can be inferred that adult volunteers are attracted to the Junior Showmanship Program because it instills positive values and positively influences the child's future. Jones et al. reported that instructor “style” was shown to have “paramount importance” for enhancing student motivation to participate (22).” Because most Juniors are not able to drive to events, success in the ring is significantly impacted by the adult's efficacy in supporting their travel needs during participation. Interactions between Juniors and their family, friends, and peers are an important component of continued commitment and assume a lower risk of dropout from the program. With these data points in mind, perhaps the addition of targeted coursework on how to effectively mentor a Junior would be a worthwhile addition to the AKC's Canine College resources.

There was a strong consensus among respondents that “working with a dog,” “feelings of accomplishment,” and “winning” were some of the more important aspects of Junior Showmanship that made it fun. This indicates that self-satisfaction and sense of fun is a primary motivator for continued participation in Junior Showmanship, which is a common theme for teens that continue participation in sport (19, 23). Under half of the respondents qualified their success in the show ring as “very high.” The simple majority of respondents fell in the minimal to moderate category. Younger participants had dramatically less success in the Best Junior competition. The length of investment required to experience this level of success leads to delayed gratification in novice participants. Survey participants clearly indicated that “success in the ring” was of high priority. The 2006 publication by Jones et al. found no significant gender difference in prioritizing “reward/status,” “situational,” or “competition” as motivational factors for participation (22). Alternatively, Kondric et al. found there to be significant differences in factors that motivate boys and girls (24); in a study of young adults in Slovenia, Croatia and Germany, males valued sport or physical activity for achieving popularity, whereas women valued it more as a means of relaxation. The display of priority by previous participants in Junior Showmanship, the majority of whom are still participating in conformation or dog sports as breeders or exhibitors, supports a correlation between “success in the ring” and continued participation. The low self-reported success rate for young Juniors who do not win should taken as a factor that needs to be addressed rather than a factor that reflects the competitive nature of the Best Junior competition. Winners from the Open Senior class are most often awarded Best Junior and winners from the Novice classes are awarded Best Junior under 5% of the time. The data show that Novice classes have the lowest number of entries, which may reflect that a lack of “success in the ring” negatively influences participation. In order to ensure a constant supply of Juniors and increase retention the data suggest that separating Best Junior competition offers more opportunity for “success in the ring” to all Juniors. A Best Junior Award for competition between the youngest Juniors and a Best Junior Award for the older Juniors allows the youngest with little or no experience to have fun and win and it allows the oldest and most experience to compete against Juniors with similar age, size and skill. This recommendation is supported by the fact that 5-years of age and experience between the youngest and oldest Juniors is not a level playing field. Also, offering opportunities to recognize achievement outside the show ring can help increase “success in the ring” among Novice class participants.

In an attempt to increase retention the AKC founded the Junior Recognition Program with the goal of rewarding Juniors for diverse engagement in AKC events. The point system recognizes participation in conformation, as well as companion and performance events. At the end of the year, Juniors that receive the most points are awarded scholarships (25). The AKC also launched the Junior Mentorship Program and Junior Ambassador Program with the goal of providing Juniors with a peer role model in the conformation ring. The older mentor benefits from teaching their younger counterpart and should be a sportsmanship role model. In turn, the young Junior has a peer who is available to answer questions about handling, husbandry, dog grooming, personal presentation, and show rules. Participation in the Junior Mentorship Program is rewarded through the Junior Ambassador Program which rewards Junior participation in all aspects of conformation and dog sports and provides clubs with resources to engage Juniors. Juniors are also granted free access to the courses and resources in the AKC Online Canine College (25). In addition to Junior programming, the AKC is working to extend its 4-H outreach, this represents an opportunity to partner with positive youth development strategies that have been developed by 4-H (2). It is recognized in many sports that early childhood experience increases the likelihood of continuing in a sport (19). Therefore, the AKC has also recently introduced the Pee Wee special attraction for children between 5 and 9 years of age. This non-competitive class was designed as a fun, introductory learning experience for both child and parent (25).

Exhibitors and breeders often articulate the belief that Juniors are “the future of the conformation and dog sport.” The validity of this credence is readily illustrated by the data collected in this survey. Despite the potential limitations of recall bias and recruitment bias, this survey demonstrates that a multitude of factors influence children participating in AKC events. Perceived achievement and adequate mentorship by adult volunteers supports continued participation in the Junior Showmanship Program and this important component can be applied across several sports and animal related programs. It may also provide guidance for development of new opportunities to engage youth in educational, sport or volunteer opportunities with animals. Furthermore, retention in the program leads to future activity as an adult exhibitor or breeder. However, the survey also indicates that dropping out of the Junior Showmanship program does not always correlate with cessation of participation in AKC events. Future studies, should included qualitative data to gain additional perspective on factors that influence participation, retention, a sense of fun and success.

Broad social factors clearly have a significant influence on continued participation in the Junior Showmanship program and future engagement, therefore it would be beneficial to prospectively study the factors related to the perceived attitudes about a lack of success in the ring to determine if they are linked to the dropout rate and/or gender discrepancies. Further study is warranted to investigate factors specifically pertaining to male participation across the animal based sport and education programs including 4-H and Pony Club. It would also be valuable to investigate how male program participants viewed their success, what aspects made the program attractive or “fun,” and how peers viewed their participation.

## Data availability statement

The original contributions presented in the study are included in the article/Supplementary material, further inquiries can be directed to the corresponding author.

## Ethics statement

Ethical review and approval was not required for the study on human participants in accordance with the local legislation and institutional requirements. Written informed consent for participation was not required for this study in accordance with the national legislation and the institutional requirements.

## Author contributions

All authors contributed to the writing of this manuscript. The survey itself was designed by CB and CO. All authors contributed to the article and approved the submitted version.

## Conflict of interest

The authors declare that the research was conducted in the absence of any commercial or financial relationships that could be construed as a potential conflict of interest.

## Publisher's note

All claims expressed in this article are solely those of the authors and do not necessarily represent those of their affiliated organizations, or those of the publisher, the editors and the reviewers. Any product that may be evaluated in this article, or claim that may be made by its manufacturer, is not guaranteed or endorsed by the publisher.
