# Supplementary material for: Retention and future involvement in the American Kennel Club Junior Showmanship Program, a youth dog breed conformation competition
Source: Front Vet Sci. 2022 Sep 23;9:871914. doi: 10.3389/fvets.2022.871914 (PMC9539916; doi:10.3389/fvets.2022.871914)
Supplement: Supplementary file 1 [file Table_1.docx]

Table 1S: Belief System Model (BSM), Dependent Variables, and control variables

|  | **BSM** | **commitment** | **Member_interest** | **AKC_recency** | **SES** | **Education** | **Judge_indif** | **Success_jr** | **Exp_fun** | **Stress_reverse** | **gender** | **age** | **Compete_date** | **scholarshp** | **Show_years** |
| --- | --- | --- | --- | --- | --- | --- | --- | --- | --- | --- | --- | --- | --- | --- | --- |
| **BSM** | **1.0** | 0.3575** | 0.4001** | 0.1438** | -0.0290 | -0.1285** | -0.2372** | 0.5275** | 0.2238** | 0.2411** | -0.0203 | -0.0645* | -0.2433** | 0.1033* | 0.1639** |
| **commitment** | 0.3575** | **1.0** | 0.7807** | 0.2272** | -0.0046 | 0.1216** | -0.0785* | 0.4648** | 0.0863** | 0.0679* | -0.626 | 0.2363** | -0.4177** | 0.0813** | 0.0048 |
| **Member interest** | 0.4001** | 0.7807** | **1.0** | 0.3087** | -0.0217 | 0.0751* | -0.0879** | 0.5220** | 0.0770* | 0.0020 | -0.1556** | 0.1118** | -0.3848** | -0.0249 | -0.0249 |
| **AKC_recency** | 0.1438** | 0.2272** | 0.3087** | **1.0** | -0.0172 | -0.0766* | -0.0072 | 0.0964** | 0.1063** | 0.0730* | -0.0682* | -0.686* | 0.0556 | 0.1012** | 0.0938** |
| **SES** | -0.0290 | -0.0046 | -0.0217 | -0.0172 | **1.0** | 0.0985** | -0.0081 | -0.0148 | 0.0107 | 0.0379 | -0.0268 | 0.0260 | 0.0193 | 0.0355 | 0.0903** |
| **Education** | -0.1285** | 0.1216** | 0.0751* | -0.0766* | 0.0985** | **1.0** | 0.0333 | -0.0282 | -0.1008 | 0.0858* | -0.0031 | 0.0195 | -0.0244 | 0.1517 | -0.0252 |
| **Judge_indif** | -0.2372** | -0.0785* | -0.0879** | -0.0072 | -0.0081 | 0.0333 | **1.0** | 0.0120 | -0.1784** | -0.2074** | 0.0011 | -0.1966** | 0.1745** | 0.0306 | 0.0160 |
| **Success_jr** | 0.5275** | 0.4648** | 0.5220** | 0.0964** | -0.0148 | -0.0282 | 0.0120 | **1.0** | 0.0340 | 0.0030 | -0.0378 | -0.1738** | -0.3311** | 0.1076** | **N/A** |
| **Exp_fun** | 0.2238** | 0.0863** | 0.0770* | 0.1063** | 0.0107 | -0.1008 | -0.1784** | 0.0340 | **1.0** | 0.1713** | -0.1503** | 0.0916* | -0.0843** | 0.035 | -0.0149 |
| **Stress_reverse** | 0.2411** | 0.0679* | 0.0020 | 0.0730* | 0.0379 | 0.0858* | -0.2074** | 0.0030 | 0.1713** | **1.0** | 0.0213 | 0.1300** | -0.1177** | -0.0116 | 0.0080 |
| **gender** | -0.0203 | -0.626 | -0.1556** | -0.0682* | -0.0268 | -0.0031 | 0.0011 | -0.0378 | -0.1503** | 0.0213 | **1.0** | **N/A** | -0.0359 | -0.0164 | -0.0064 |
| **Age** | -0.0645* | 0.2363** | 0.1118** | -0.0686* | 0.0260 | 0.0195 | -0.1966** | -0.1738** | 0.0916* | 0.1300** | **N/A** | **1.0** | **N/A** | -0.2324** | -0.1418** |
| **Compete_date** | -0.2433** | -0.4177** | -0.3848** | 0.0556 | 0.0193 | -0.0244 | 0.1745** | -0.3311** | -0.0843** | -0.1177** | -0.0359 | **N/A** | **1.0** | 0.2344 | 0.1266** |
| **scholarship** | 0.1033* | 0.0813** | 0.0811* | 0.1012** | 0.0355 | 0.1517 | 0.0306 | 0.1076** | 0.035 | -0.0116 | -0.0164 | -0.2324** | 0.2344 | **1.0** | 0.0864** |
| **Show_years** | 0.1639** | 0.0048 | -0.0249 | 0.0938** | 0.0903** | -0.0252 | 0.0160 | N/A | -0.0149 | 0.0080 | -0.0064 | -0.1418** | 0.1266** | 0.0864** | **1.0** |

** highly significant p=0.01 *significant p=0.05

highly correlated r=1.0-0.07 – moderately correlated r= 0.6-0.4 – weakly correlated r=0.3-0.01

Legend:

Belief System Model (BSM): This measures the camaraderie amongst the juniors, based on: their supporting network, friends reaction, how friendly the judges were, how much guidance the judges provided, how many friends at show, if they were attracted to the AKC because: their friend was a junior, if their parent encouraged them, or if they were attracted by someone else (alpha= 0.6267 very stable)

Commitment: Measures the current commitment to the AKC based on the number of meetings attended, or served as an officer, AKC delegate, AKC judge and AKC eligibility. (alpha=0.7074- very stable)

Member Interest: Measures current interest in the AKC based on the number of memberships to clubs and their AKC eligibility. Clubs include: parent club, merit program, all breed member, and other dog club. The higher the number, the more involved they currently are in the AKC. (alpha=0.5684- moderately stable).

AKC Recency: This measures the extent of your recent involvement in AKC shows, based on: the number of dogs handled in conformation in the last 12 months and the number of champions finished and handled in the last 5 years. (The higher the score the more active they have been within the past 5 years) (alpha =0.8637- very stable)

SES = socio-economic status,

Education: Highest level of education received, the higher the number, the higher their education level.

Judge Indif = Judges’ Indifference

Success_Jr = Success as a Junior: defined as one that spends at least 3 years in the show ring and were aged 15-18 when they last entered the show ring.

Exp-fun = Experience in the show ring (how fun?)

Stress – reverse = Experience in the ring was NOT stressful: any positive correlation means that the experience in the ring not being stressful

Scholarship: Did they receive a scholarship as an AKC junior
