# Supplementary material for: Retention and future involvement in the American Kennel Club Junior Showmanship Program, a youth dog breed conformation competition
Source: Front Vet Sci. 2022 Sep 23;9:871914. doi: 10.3389/fvets.2022.871914 (PMC9539916; doi:10.3389/fvets.2022.871914)
Supplement: Supplementary file 2 [file Table_2.docx]

Table 2S: What affects BSM and how does it affect involvement as a jr, involvement now and dedication to the sport

|  | **BSM** | Quality of life | Breeder | Exhibitors | Siblings | Friends | dedication | Involvement jr | Involvement_ now |
| --- | --- | --- | --- | --- | --- | --- | --- | --- | --- |
| **BSM** | **1.0** | 0.1984** | 0.4982** | 0.5112** | 0.2507** | N/A | 0.3064** | 0.4290** | 0.1533** |
| Dogs quality of life | 0.1984** | **1.0** | -0.0138 | -0.0635 | 0.0006 | 0.1292** | 0.1156** | 0.0712* | 0.1159** |
| Breeders | 0.4982** | -0.0138 | **1.0** | 0.8020** | 0.2442** | 0.2057** | 0.3614** | 0.3304** | -0.0029 |
| Exhibitors | 0.5112** | -0.0635 | 0.8020** | **1.0** | 0.2281** | 0.1965** | 0.3370** | 0.3544** | 0.0103 |
| Siblings | 0.2507** | 0.0006 | 0.2442** | 0.2281** | **1.0** | 0.1742** | 0.1643** | 0.0490 | -0.0665 |
| Friends | N/A | 0.1292** | 0.2057** | 0.1965** | 0.1742** | **1.0** | 0.3027** | 0.282 | 0.202 |
| Dedication | 0.3064** | 0.1156** | 0.3614** | 0.3370** | 0.1643** | 0.3027** | **1.0** | 0.0416 | -0.0454 |
| Involvement_jr | 0.4290** | 0.0712* | 0.3304** | 0.3544** | 0.0490 | 0.282 | 0.0416 | **1.0** | 0.366** |
| Involvement_now | 0.1533** | 0.1159** | -0.0029 | 0.0103 | -0.0665 | 0.202 | -0.0454 | 0.366** | **1.0** |

*p≤0.05 **p≤0.001

*highly significant p=0.01 – significant p=0.05

* highly correlated r=1.0-0.07 – moderately correlated r= 0.6-0.4 – weakly correlated r=0.3-0.01
