# Supplementary material for: Retention and future involvement in the American Kennel Club Junior Showmanship Program, a youth dog breed conformation competition
Source: Front Vet Sci. 2022 Sep 23;9:871914. doi: 10.3389/fvets.2022.871914 (PMC9539916; doi:10.3389/fvets.2022.871914)
Supplement: Supplementary file 3 [file Data_Sheet_1.PDF]

AKC Junior Survey

1. I competed as an AKC junior between

	Response Percent	Response Count
2002-2012	24.4%	329
**1992-2001**	**34.5%**	**464**
1982-1991	18.1%	243
1972-1981	12.7%	171
1962-1971	7.8%	105
1952-1961	1.8%	24
before 1952	0.7%	10
	**answered question**	**1,346**
	**skipped question**	**0**

2. I am a

	Response Percent	Response Count
male	15.1%	131
**female**	**84.9%**	**735**
	**answered question**	**866**
	**skipped question**	**480**

3. My current age is

	Response Average	Response Total	Response Count
**years**	**39.32**	**34,050**	866
		**answered question**	**866**
		**skipped question**	**480**

4. My current or previous jobs related to dogs include (mark all that apply)

	Response Percent	Response Count
Veterinary medicine or other animal medical field	20.1%	174
Animal Research	4.2%	36
Dog Food Industry	3.8%	33
AKC	6.9%	60
**Breeder/Exhibitor**	**75.6%**	**655**
Professional Handler	35.6%	308
Animal Advertising Publicity	1.7%	15
Animal Law	1.0%	9
Trainer	28.5%	247
No job related to dogs	16.4%	142
	Other (please specify)	204
	**answered question**	**866**
	**skipped question**	**480**

5. My current occupation is

	Response Count
	866
**answered question**	**866**
**skipped question**	**480**

6. My most advanced education was

	Response Percent	Response Count
did not complete high school	0.6%	5
High school or GED	22.9%	198
2 year college degree	20.6%	178
**4 year college degree**	**37.2%**	**322**
Master's degree	13.2%	114
Doctorate	5.7%	49
specify advanced degree		156
**answered question**		**866**
**skipped question**		**480**

7. I received an AKC Junior Scholarship or club scholarship

		Response Percent	Response Count
yes		11.9%	103
**no**		**88.1%**	**763**
		**answered question**	**866**
		**skipped question**	**480**

8. I believe dogs contribute to the quality of my life through

	strongly agree	agree	neutral	disagree	strongly disagree	Rating Average	Response Count
companionship	**94.1% (815)**	4.7% (41)	0.6% (5)	0.1% (1)	0.5% (4)	1.08	866
service	**51.8% (425)**	27.3% (224)	19.1% (157)	1.0% (8)	0.9% (7)	1.72	821
research	27.8% (225)	20.6% (167)	**38.3% (310)**	6.9% (56)	6.3% (51)	2.43	809
						Other (please specify)	59
						**answered question**	**866**
						**skipped question**	**480**

9. If yes, how many years of scholarship support?

	Response Percent	Response Count
**1**	**41.7%**	**43**
2	24.3%	25
3	11.7%	12
4	16.5%	17
>4	5.8%	6
	**answered question**	**103**
	**skipped question**	**1,243**

10. I owned my first dog when I was

	Response Percent	Response Count
**1**	**22.4%**	**190**
2	2.5%	21
3	3.5%	30
4	3.8%	32
5	8.8%	75
6	6.3%	53
7	5.9%	50
8	9.2%	78
9	9.1%	77
10	8.6%	73
11	4.6%	39
12	5.2%	44
13	3.7%	31
14	2.6%	22
15	1.3%	11
16	1.1%	9
17	0.2%	2
18	0.1%	1
>18	1.1%	9
never	0.1%	1
	**answered question**	**848**
	**skipped question**	**498**

11. My parents were

	Response Percent	Response Count
Breeders	51.5%	437
**Exhibitors**	**52.7%**	**447**
Professional Handlers	9.2%	78
None of the above	40.6%	344
**answered question**		**848**
**skipped question**		**498**

12. I had siblings in the Junior program

	Response Percent	Response Count
yes	32.7%	277
**no**	**67.3%**	**571**
**answered question**		**848**
**skipped question**		**498**

13. I had friends in the Junior program

	Response Percent	Response Count
**yes**	**78.6%**	**663**
no	21.4%	181
**answered question**		**844**
**skipped question**		**502**

14. The first class I entered as a Junior was

	Response Percent	Response Count
**Novice Jr**	**78.2%**	**663**
Novice Intermediate	4.0%	34
Novice Sr	15.8%	134
Open Jr	1.4%	12
Open Intermediate	0.2%	2
Open Sr	0.7%	6
	Other (please specify)	46
	**answered question**	**848**
	**skipped question**	**498**

15. The last class I entered as a Junior was

	Response Percent	Response Count
Novice Jr	2.4%	20
Novice Intermediate	1.3%	11
Novice Sr	3.2%	27
Open Jr	5.0%	42
Open Intermediate	2.5%	21
**Open Sr**	**86.8%**	**736**
	Other (please specify)	36
	**answered question**	**848**
	**skipped question**	**498**

16. My age in years when I was last in the show ring as a Junior

		Response Percent	Response Count
9		0.2%	2
10		0.7%	6
11		0.8%	7
12		2.0%	17
13		2.9%	25
14		2.8%	24
15		5.7%	48
16		15.4%	131
17		32.4%	275
**18**		**36.7%**	**311**
never		0.2%	2
		**answered question**	**848**
		**skipped question**	**498**

17. I spent ___ years in the show ring as a Junior

	Response Percent	Response Count
<1	1.7%	14
1	1.9%	16
2	6.4%	54
3	8.3%	70
4	12.5%	106
5	12.0%	102
6	12.1%	103
7	13.1%	111
**8**	**18.9%**	**160**
9	13.2%	112
	**answered question**	**848**
	**skipped question**	**498**

18. I showed ____ breeds

	Response Percent	Response Count
none	0.0%	0
**1**	**32.1%**	**263**
2	23.0%	189
3	15.0%	123
4	7.6%	62
>4	22.3%	183
	**answered question**	**820**
	**skipped question**	**526**

19. My breed(s) as a Junior were

	Response Percent	Response Count
**first**	**100.0%**	**820**
second	67.7%	555
third	42.6%	349
fourth	26.2%	215
	**answered question**	**820**
	**skipped question**	**526**

20. As a Junior I competed in: (mark all that apply)

	Response Percent	Response Count
**Junior Showmanship**	**98.8%**	**810**
Obedience	41.3%	339
Breed Competition	90.1%	739
Rally	2.4%	20
Agility	6.3%	52
Performance	6.7%	55
	Other (please specify)	51
	**answered question**	**820**
	**skipped question**	**526**

21. As a Junior I showed

	Response Percent	Response Count
**My own dog**	**86.2%**	**707**
My family's dog	62.3%	511
Other people's dogs	44.6%	366
	**answered question**	**820**
	**skipped question**	**526**

22. As a Junior I showed (check all that apply)

	Response Percent	Response Count
**intact female(s)**	**85.5%**	**701**
spayed female(s)	16.5%	135
intact male(s)	85.1%	698
neutered male(s)	12.3%	101
	**answered question**	**820**
	**skipped question**	**526**

23. As a Junior I finished ____ dogs

	Response Percent	Response Count
0	24.4%	200
1	11.2%	92
2	12.2%	100
3	6.7%	55
4	4.9%	40
**>4**	**40.6%**	**333**
	**answered question**	**820**
	**skipped question**	**526**

24. As a Junior, I bred ____ litters.

	Response Percent	Response Count
**none**	**45.6%**	**374**
1	14.8%	121
2	14.9%	122
3	6.7%	55
4	2.3%	19
>4	15.7%	129
	**answered question**	**820**
	**skipped question**	**526**

25. During my Junior competition years, I lived in a ________ (check all that apply)

	Response Percent	Response Count
rural area	40.0%	328
**suburban area**	**55.4%**	**454**
urban area	10.9%	89
	**answered question**	**820**
	**skipped question**	**526**

26. During my Junior competition years, I lived in a ________ (check all that apply)

	Response Percent	Response Count
**single family dwelling**	**97.0%**	**795**
multifamily dwelling	2.3%	19
apartment/condo	1.6%	13
	**answered question**	**820**
	**skipped question**	**526**

27. During my Junior competition years, my dog typically had access to (check all that apply)

	Response Percent	Response Count
**a fenced yard**	**91.3%**	**749**
free range of our property (unfenced)	14.4%	118
public parks	21.6%	177
leash walks	54.6%	448
	**answered question**	**820**
	**skipped question**	**526**

28. I was attracted to the Junior program because (check all that apply)

		Response Percent	Response Count
saw others do it		47.4%	389
had a friend who was a Jr.		22.4%	184
was encouraged by parents		50.0%	410
**traveled to shows with parents and friends and liked the idea**		**66.5%**	**545**
		Other (please specify)	144
		**answered question**	**820**
		**skipped question**	**526**

29. My friends thought my involvement as a Junior handler was

	very cool	indifferent	not cool	they didn't know I was a Jr	Rating Average	Response Count
reaction	32.6% (267)	**47.3% (388)**	8.2% (67)	12.0% (98)	2.00	820
					**answered question**	**820**
					**skipped question**	**526**

30. Rate how each of the following made Jr. handling fun:

	strongly agree	agree	indifferent	disagree	strongly disagree	not applicable	Rating Average	Res[illegible] C[illegible]
working with a dog	**85.8% (702)**	13.7% (112)	0.5% (4)	0.0% (0)	0.0% (0)	0.0% (0)	1.15	
friends at the show	**49.9% (406)**	29.9% (243)	15.6% (127)	2.3% (19)	0.9% (7)	1.5% (12)	1.79	
feelings of accomplishment	**77.5% (633)**	19.3% (158)	2.3% (19)	0.6% (5)	0.1% (1)	0.1% (1)	1.27	
my dog was easy to train	16.0% (130)	**28.6% (233)**	20.6% (168)	26.5% (216)	7.2% (59)	1.0% (8)	2.83	
my dog was a challenge	30.8% (248)	**39.3% (316)**	14.9% (120)	10.6% (85)	2.7% (22)	1.7% (14)	2.20	
winning was fun	**63.5% (517)**	30.7% (250)	4.5% (37)	0.5% (4)	0.1% (1)	0.6% (5)	1.45	
opportunity to watch professional handler's	**49.1% (400)**	25.6% (209)	17.2% (140)	4.2% (34)	2.6% (21)	1.3% (11)	1.90	
							**answered question**	
							**skipped question**	

31. My experience in the show ring was

	very	indifferent	not at all	N/A	Rating Average	Response Count
fun	**92.2% (756)**	6.6% (54)	1.2% (10)	0.0% (0)	1.09	820
stressful	26.0% (213)	**41.1% (337)**	31.7% (260)	1.2% (10)	2.06	820
					**answered question**	**820**
					**skipped question**	**526**

32. My success in the show ring was

	Response Percent	Response Count
very high	44.5%	365
**moderate**	**47.7%**	**391**
minimal	7.3%	60
none	0.4%	3
not applicable	0.1%	1
	**answered question**	**820**
	**skipped question**	**526**

33. The judges were

	strongly agree	agree	neutral	disagree	strongly disagree	not applicable	Rating Average	Respon Coun
friendly	25.0% (205)	**52.4% (430)**	17.4% (143)	4.4% (36)	0.7% (6)	0.0% (0)	2.03	
provided guidance	19.3% (158)	**37.6% (308)**	27.0% (221)	13.2% (108)	2.9% (24)	0.1% (1)	2.43	
businesslike	20.6% (169)	**59.6% (489)**	18.0% (148)	0.9% (7)	0.7% (6)	0.1% (1)	2.02	
indifferent	4.4% (36)	25.0% (205)	**39.1% (321)**	22.9% (188)	6.1% (50)	2.4% (20)	3.09	
							**answered question**	
							**skipped question**	

34. The following people served as mentors during my Junior program

	very much	some	not at all	not relavant	Rating Average	Response Count
Mother	**53.7% (423)**	25.3% (199)	17.0% (134)	4.1% (32)	1.71	788
Father	20.6% (158)	26.1% (200)	**40.9% (313)**	12.4% (95)	2.45	766
Sibling	9.4% (69)	14.9% (109)	**47.7% (350)**	28.0% (205)	2.94	733
Friend	**47.1% (369)**	34.5% (270)	12.5% (98)	5.9% (46)	1.77	783
Judge	20.5% (154)	36.1% (271)	**36.4% (273)**	7.1% (53)	2.30	751
Professional Handler	**46.3% (367)**	29.8% (236)	19.1% (151)	4.8% (38)	1.82	792
					**answered question**	**820**
					**skipped question**	**526**

35. I own (mark all that apply)

	none	1	2	3	4	>5	Response Count
purebred dog(s)	5.0% (40)	7.9% (64)	12.9% (104)	9.6% (77)	11.7% (94)	**53.0% (427)**	806
mixed breed dog(s)	**85.0% (551)**	10.8% (70)	2.3% (15)	1.1% (7)	0.2% (1)	0.6% (4)	648
cat(s)	**51.6% (362)**	23.1% (162)	14.5% (102)	5.0% (35)	3.3% (23)	2.6% (18)	702
bird(s)	**86.1% (570)**	6.5% (43)	3.2% (21)	0.9% (6)	0.6% (4)	2.7% (18)	662
fish	**78.2% (512)**	4.1% (27)	3.2% (21)	2.3% (15)	0.6% (4)	11.6% (76)	655
other animals	**72.4% (458)**	7.7% (49)	6.2% (39)	2.7% (17)	1.9% (12)	9.2% (58)	633
						Other (please specify)	163
						**answered question**	**811**
						**skipped question**	**535**

36. The breeds of dog I own now are

	Response Percent	Response Count
if none enter N/A	20.8%	169
**first**	**86.9%**	**705**
second	58.7%	476
third	26.8%	217
fourth	10.4%	84
	**answered question**	**811**
	**skipped question**	**535**

37. I have bred ___ litter(s) in the past 5 years

	Response Percent	Response Count
**none**	**32.3%**	**262**
1	13.1%	106
2	16.3%	132
3	10.2%	83
4	9.1%	74
>4	19.0%	154
	**answered question**	**811**
	**skipped question**	**535**

38. I have bred __ champions in the last 5 years

	Response Percent	Response Count
none	13.1%	72
1	8.6%	47
2	12.0%	66
3	12.4%	68
4	8.6%	47
**>4**	**45.3%**	**248**
	**answered question**	**548**
	**skipped question**	**798**

39. Are you in the Breeder of Merit program?

	Response Percent	Response Count
yes	37.2%	204
**no**	**62.8%**	**344**
	**answered question**	**548**
	**skipped question**	**798**

40. Unless I intend to breed them, I prefer my dogs to be spayed or neutered

	Response Percent	Response Count
**strongly agree**	**38.0%**	**308**
agree	29.8%	241
neutral	19.9%	161
disagree	8.3%	67
strongly disagree	4.1%	33
	**answered question**	**810**
	**skipped question**	**536**

41. Are you currently eligible to compete in AKC events?

	Response Percent	Response Count
**yes**	**97.9%**	**793**
no	2.1%	17
	**answered question**	**810**
	**skipped question**	**536**

42. I have handled ____ number of dogs in conformation in the last 12 months

	Response Percent	Response Count
none	17.3%	137
1	9.2%	73
2	11.0%	87
3	6.7%	53
4	5.4%	43
5	3.8%	30
**>5**	**46.7%**	**370**
	**answered question**	**793**
	**skipped question**	**553**

43. In the past 12 months I have handled dog(s) in: (mark all that apply)

	Response Percent	Response Count
Obedience	18.8%	149
Agility	10.2%	81
Rally	18.3%	145
Hunting	8.8%	70
Tracking	2.6%	21
Lure coursing	8.2%	65
Working dog sport	4.8%	38
**other**	**35.9%**	**285**
am not involved	33.0%	262
	Other (please specify)	278
	**answered question**	**793**
	**skipped question**	**553**

44. I have handled and finished _____ champions in the last 5 years

	Response Percent	Response Count
none	23.3%	185
1	9.5%	75
2	10.3%	82
3	6.6%	52
4	5.3%	42
**>4**	**46.2%**	**366**
	**answered question**	**793**
	**skipped question**	**553**

45. While eligible I have handled ____ number of dogs in conformation

	Response Percent	Response Count
none	12.5%	2
1	6.3%	1
2	0.0%	0
3	12.5%	2
4	0.0%	0
5	6.3%	1
**>5**	**62.5%**	**10**
	**answered question**	**16**
	**skipped question**	**1,330**

46. While eligible I have handled dog(s) in: (mark all that apply)

	Response Percent	Response Count
**Obedience**	**37.5%**	**6**
Agility	12.5%	2
Rally	0.0%	0
Hunting	0.0%	0
Tracking	6.3%	1
Lure coursing	12.5%	2
Working dog sport	6.3%	1
other	12.5%	2
**am not involved**	**37.5%**	**6**
	Other (please specify)	2
	**answered question**	**16**
	**skipped question**	**1,330**

47. While eligible I have handled and finished _____ champions

	Response Percent	Response Count
none	25.0%	4
1	12.5%	2
2	0.0%	0
3	0.0%	0
4	0.0%	0
**>4**	**62.5%**	**10**
	**answered question**	**16**
	**skipped question**	**1,330**

48. I am a member of (check all that apply)

	Response Percent	Response Count
**Parent club**	**66.1%**	**532**
All-breed club	38.5%	310
Other dog club	37.1%	299
No dog related organizations	19.3%	155
	Other (please specify)	144
	**answered question**	**805**
	**skipped question**	**541**

49. In the past 2 years, I have attended meetings at (mark all that apply)

	Response Percent	Response Count
Dog training club	24.1%	194
**Specialty breed club**	**58.5%**	**471**
All-Breed club	40.4%	325
4H club	11.1%	89
other	4.7%	38
none of the above	23.1%	186
	Other (please specify)	33
	**answered question**	**805**
	**skipped question**	**541**

50. In the past 5 years, I have served as a (mark all that apply)

	Response Percent	Response Count
Dog club officer	38.1%	307
AKC delegate	1.9%	15
Junior Showmanship Judge	21.9%	176
Conformation Judge	15.2%	122
Judge of other dog events	20.4%	164
**None of the above**	**41.9%**	**337**
	**answered question**	**805**
	**skipped question**	**541**
