# Supplementary material for: Retention and future involvement in the American Kennel Club Junior Showmanship Program, a youth dog breed conformation competition
Source: Front Vet Sci. 2022 Sep 23;9:871914. doi: 10.3389/fvets.2022.871914 (PMC9539916; doi:10.3389/fvets.2022.871914)
Supplement: Supplementary file 4 [file Data_Sheet_2.PDF]

Introduction

PURPOSE STATEMENT
There is little data or evidence that explains the usefulness and value of the AKC junior Showmanship program. No long term data has been collected that supports the belief that juniors are the future of the sport. Although many clubs support and offer a junior program no evidence has been collected that demonstrates that these efforts produce future breeders, handlers or exhibitors. Given the lack of longitudinal data the following survey was developed as a joint effort by Dr. Carmen Battaglia PhD of the AKC and Dr. Cindy Otto DVM, University of Pennsylvania, School of Veterinary Medicine.
For purposes of this survey, the investigators are interested in juniors who were in competition 10 or more years ago as a junior handler. This survey will attempt to identify characteristics that are associated with young people who became interested in AKC junior showmanship program. A secondary question focuses on identifying the factors that attracted them to the dog world of juniors and why some continued or fail to pursue their interest in the dog sport.

By completing this survey, you acknowledge that you are giving your consent to use the information that you provide for research purposes. You recognize that participation is entirely voluntary. If you have any research related questions or concerns you are able to contact Dr. Cynthia Otto at cmotto@vet.upenn.edu. For programmatic questions related to the Junior Handler Program or the AKC you can contact Dr. Carmen Battaglia at cbattaglia@mindspring.com.

WHO SHOULD COMPLETE THIS SURVEY?

This survey is designed to be completed by any junior who was in competition prior to 2000. All parts of the survey must be completed to be included in our analysis. Some questions have more than one answer. If you know of a junior who competed prior to 2000 please forward the link to them.

*1. I competed as an AKC junior between

- ◯ 2002-2012
- ◯ 1992-2001
- ◯ 1982-1991
- ◯ 1972-1981
- ◯ 1962-1971
- ◯ 1952-1961
- ◯ before 1952

About You

***2. I am a**

- ◯ male
- ◯ female

***3. My current age is**

years ______

***4. My current or previous jobs related to dogs include (mark all that apply)**

- ☐ Veterinary medicine or other animal medical field
- ☐ Animal Research
- ☐ Dog Food Industry
- ☐ AKC
- ☐ Breeder/Exhibitor
- ☐ Professional Handler
- ☐ Animal Advertising Publicity
- ☐ Animal Law
- ☐ Trainer
- ☐ No job related to dogs

Other (please specify)

______

***5. My current occupation is**

______

***6. My most advanced education was**

- ◯ did not complete high school
- ◯ High school or GED
- ◯ 2 year college degree
- ◯ 4 year college degree
- ◯ Master's degree
- ◯ Doctorate

specify advanced degree

______

***7. I received an AKC Junior Scholarship or club scholarship**

- ◯ yes
- ◯ no

* 8. I believe dogs contribute to the quality of my life through

	strongly agree	agree	neutral	disagree	strongly disagree
companionship	◯	◯	◯	◯	◯
service	◯	◯	◯	◯	◯
research	◯	◯	◯	◯	◯

Other (please specify)

scholarship info

***9. If yes, how many years of scholarship support?**

- ◯ 1
- ◯ 2
- ◯ 3
- ◯ 4
- ◯ >4

*** 10. I owned my first dog when I was**

- ○ 1
- ○ 2
- ○ 3
- ○ 4
- ○ 5
- ○ 6
- ○ 7
- ○ 8
- ○ 9
- ○ 10
- ○ 11
- ○ 12
- ○ 13
- ○ 14
- ○ 15
- ○ 16
- ○ 17
- ○ 18
- ○ >18
- ○ never

*** 11. My parents were**

- ☐ Breeders
- ☐ Exhibitors
- ☐ Professional Handlers
- ☐ None of the above

*** 12. I had siblings in the Junior program**

- ○ yes
- ○ no

13. I had friends in the Junior program

- ○ yes
- ○ no

*** 14. The first class I entered as a Junior was**

- ☐ Novice Jr
- ☐ Novice Intermediate
- ☐ Novice Sr
- ☐ Open Jr
- ☐ Open Intermediate
- ☐ Open Sr

Other (please specify)

*15. The last class I entered as a Junior was

- ☐ Novice Jr
- ☐ Novice Intermediate
- ☐ Novice Sr
- ☐ Open Jr
- ☐ Open Intermediate
- ☐ Open Sr

Other (please specify)

*16. My age in years when I was last in the show ring as a Junior

- ◯ 9
- ◯ 10
- ◯ 11
- ◯ 12
- ◯ 13
- ◯ 14
- ◯ 15
- ◯ 16
- ◯ 17
- ◯ 18
- ◯ never

*17. I spent ___ years in the show ring as a Junior

- ◯ <1
- ◯ 1
- ◯ 2
- ◯ 3
- ◯ 4
- ◯ 5
- ◯ 6
- ◯ 7
- ◯ 8
- ◯ 9

My experience as a Junior

Please respond to the following questions related to the time you were competing as a Junior

*18. I showed ____ breeds

- ◯ none
- ◯ 1
- ◯ 2
- ◯ 3
- ◯ 4
- ◯ >4

*19. My breed(s) as a Junior were

first

second

third

fourth

*20. As a Junior I competed in: (mark all that apply)

- ☐ Junior Showmanship
- ☐ Obedience
- ☐ Breed Competition
- ☐ Rally
- ☐ Agility
- ☐ Performance

Other (please specify)

*21. As a Junior I showed

- ☐ My own dog
- ☐ My family's dog
- ☐ Other people's dogs

*22. As a Junior I showed (check all that apply)

- ☐ intact female(s)
- ☐ spayed female(s)
- ☐ intact male(s)
- ☐ neutered male(s)

***23. As a Junior I finished ___ dogs**

- ◯ 0
- ◯ 1
- ◯ 2
- ◯ 3
- ◯ 4
- ◯ >4

***24. As a Junior, I bred ___ litters.**

- ◯ none
- ◯ 1
- ◯ 2
- ◯ 3
- ◯ 4
- ◯ >4

***25. During my Junior competition years, I lived in a _______ (check all that apply)**

- ☐ rural area
- ☐ suburban area
- ☐ urban area

***26. During my Junior competition years, I lived in a _______ (check all that apply)**

- ☐ single family dwelling
- ☐ multifamily dwelling
- ☐ apartment/condo

***27. During my Junior competition years, my dog typically had access to (check all that apply)**

- ☐ a fenced yard
- ☐ free range of our property (unfenced)
- ☐ public parks
- ☐ leash walks

***28. I was attracted to the Junior program because (check all that apply)**

- ☐ saw others do it
- ☐ had a friend who was a Jr.
- ☐ was encouraged by parents
- ☐ traveled to shows with parents and friends and liked the idea

Other (please specify)

***29. My friends thought my involvement as a Junior handler was**

	very cool	indifferent	not cool	they didn't know I was a Jr
reaction	◯	◯	◯	◯

30. Rate how each of the following made Jr. handling fun:

	strongly agree	agree	indifferent	disagree	strongly disagree	not applicable
working with a dog	◯	◯	◯	◯	◯	◯
friends at the show	◯	◯	◯	◯	◯	◯
feelings of accomplishment	◯	◯	◯	◯	◯	◯
my dog was easy to train	◯	◯	◯	◯	◯	◯
my dog was a challenge	◯	◯	◯	◯	◯	◯
winning was fun	◯	◯	◯	◯	◯	◯
opportunity to watch professional handler's	◯	◯	◯	◯	◯	◯

***31. My experience in the show ring was**

	very	indifferent	not at all	N/A
fun	◯	◯	◯	◯
stressful	◯	◯	◯	◯

***32. My success in the show ring was**

◯ very high ◯ moderate ◯ minimal ◯ none ◯ not applicable

***33. The judges were**

	strongly agree	agree	neutral	disagree	strongly disagree	not applicable
friendly	◯	◯	◯	◯	◯	◯
provided guidance	◯	◯	◯	◯	◯	◯
businesslike	◯	◯	◯	◯	◯	◯
indifferent	◯	◯	◯	◯	◯	◯

34. The following people served as mentors during my Junior program

	very much	some	not at all	not relavant
Mother	◯	◯	◯	◯
Father	◯	◯	◯	◯
Sibling	◯	◯	◯	◯
Friend	◯	◯	◯	◯
Judge	◯	◯	◯	◯
Professional Handler	◯	◯	◯	◯

My current AKC or dog sport involvement

*35. I own (mark all that apply)

	none	1	2	3	4	>5
purebred dog(s)	○	○	○	○	○	○
mixed breed dog(s)	○	○	○	○	○	○
cat(s)	○	○	○	○	○	○
bird(s)	○	○	○	○	○	○
fish	○	○	○	○	○	○
other animals	○	○	○	○	○	○

Other (please specify)

*36. The breeds of dog I own now are

if none enter N/A

first

second

third

fourth

*37. I have bred ___ litter(s) in the past 5 years

- ○ none
- ○ 1
- ○ 2
- ○ 3
- ○ 4
- ○ >4

***38. I have bred __ champions in the last 5 years**

- ◯ none
- ◯ 1
- ◯ 2
- ◯ 3
- ◯ 4
- ◯ >4

***39. Are you in the Breeder of Merit program?**

- ◯ yes
- ◯ no

***40. Unless I intend to breed them, I prefer my dogs to be spayed or neutered**

- ◯ strongly agree
- ◯ agree
- ◯ neutral
- ◯ disagree
- ◯ strongly disagree

***41. Are you currently eligible to compete in AKC events?**

- ◯ yes
- ◯ no

*42. I have handled ___ number of dogs in conformation in the last 12 months

- ◯ none
- ◯ 1
- ◯ 2
- ◯ 3
- ◯ 4
- ◯ 5
- ◯ >5

*43. In the past 12 months I have handled dog(s) in: (mark all that apply)

- ☐ Obedience
- ☐ Agility
- ☐ Rally
- ☐ Hunting
- ☐ Tracking
- ☐ Lure coursing
- ☐ Working dog sport
- ☐ other
- ☐ am not involved

Other (please specify)

*44. I have handled and finished ____ champions in the last 5 years

- ☐ none
- ☐ 1
- ☐ 2
- ☐ 3
- ☐ 4
- ☐ >4

***45. While eligible I have handled ___ number of dogs in conformation**

- ◯ none
- ◯ 1
- ◯ 2
- ◯ 3
- ◯ 4
- ◯ 5
- ◯ >5

***46. While eligible I have handled dog(s) in: (mark all that apply)**

- ☐ Obedience
- ☐ Agility
- ☐ Rally
- ☐ Hunting
- ☐ Tracking
- ☐ Lure coursing
- ☐ Working dog sport
- ☐ other
- ☐ am not involved

Other (please specify)

***47. While eligible I have handled and finished ____ champions**

- ☐ none
- ☐ 1
- ☐ 2
- ☐ 3
- ☐ 4
- ☐ >4

***48. I am a member of (check all that apply)**

- ☐ Parent club
- ☐ All-breed club
- ☐ Other dog club
- ☐ No dog related organizations

Other (please specify)

*49. In the past 2 years, I have attended meetings at (mark all that apply)

- [ ] Dog training club
- [ ] Specialty breed club
- [ ] All-Breed club
- [ ] 4H club
- [ ] other
- [ ] none of the above

Other (please specify)

*50. In the past 5 years, I have served as a (mark all that apply)

- [ ] Dog club officer
- [ ] AKC delegate
- [ ] Junior Showmanship Judge
- [ ] Conformation Judge
- [ ] Judge of other dog events
- [ ] None of the above

TIME PERIOD FOR THIS SURVEY:

All surveys must be received no later than _______________

REPORTING

A copy to the report and analysis will be posted on the site. WWW.Breedingbetterdogs.com
